# 1-(Benz­yloxy)naphthalene

**DOI:** 10.1107/S1600536811025219

**Published:** 2011-07-06

**Authors:** Perumal Venkatesan, S. Arunadevi, M. I. Fazal Mohamed, Andivelu Ilangovan

**Affiliations:** a School of Chemistry, Bharathidasn University, Tiruchirappalli 620 024, Tamilnadu, India; b PG and Research Department of Chemistry, Urumu Dhanalakshmi College, Tiruchirappalli 620 019, Tamilnadu, India; c PG and Research Department of Chemistry, Jamal Mohamed College (Autonomous), Tiruchirappalli 620 020, Tamilnadu, India

## Abstract

In the title compound, C_17_H_14_O, the dihedral angle between the naphthyl ring system and the benzyl group is 83.22 (4)°. Both of these moieties are planar, with mean deviations from their least-squares planes, defined by the naphthyl ring C atoms and the O atom, and the phenyl ring C atoms and the benzyl α-C atom, of 0.0176 (1) and 0.0024 (13) Å, respectively. The crystal structure is stabilized by C—H⋯π and π–π inter­actions [centroid–centroid distance = 3.7817 (10) Å].

## Related literature

For the synthesis of benzyl-1-naphthyl ether, see: Mohamed & Arunadevi (2010 ▶); For related structures, see: Hassan *et al.* (2008*a* ▶,*b* ▶,*c* ▶,*d* ▶, 2009*a* ▶,*b* ▶); Abdullah & Ng (2008 ▶). For applications of naphthyl ethers, see: Fernandes *et al.* (2011 ▶); Scanu *et al.* (2007 ▶); He *et al.* (2008 ▶). For the use of benzyl protecting groups, see: Rao & Senthilkumar (2001 ▶). For the role of benzyl ether inter­mediates in sigmatropic rearrangement reactions, see: Salunkhe *et al.* (1994 ▶).
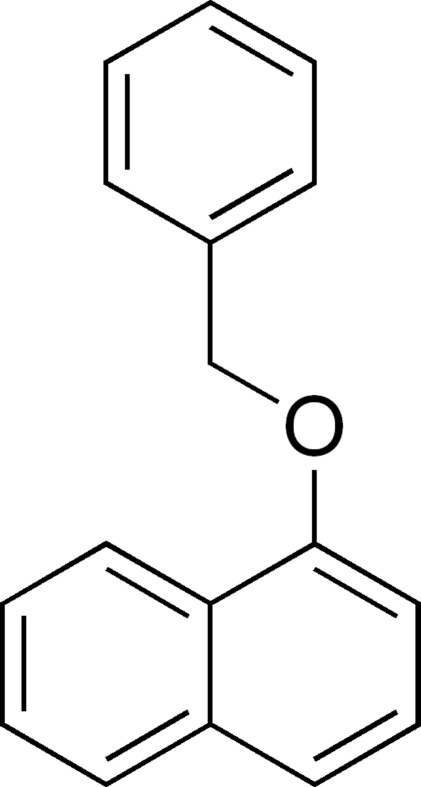


## Experimental

### 

#### Crystal data

C_17_H_14_O
*M* *_r_* = 234.28
Orthorhombic, 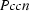

*a* = 13.0210 (6) Å
*b* = 24.8832 (10) Å
*c* = 7.9478 (3) Å
*V* = 2575.12 (19) Å^3^
*Z* = 8
Mo *K*α radiation
μ = 0.07 mm^−1^
*T* = 296 K
0.07 × 0.06 × 0.06 mm

#### Data collection

Bruker SMART CCD area-detector diffractometer
22084 measured reflections
2418 independent reflections
1609 reflections with *I* > 2σ(*I*)
*R*_int_ = 0.059

#### Refinement

*R*[*F* ^2^ > 2σ(*F* ^2^)] = 0.039
*wR*(*F* ^2^) = 0.099
*S* = 1.02
2418 reflections
164 parameters
H-atom parameters constrained
Δρ_max_ = 0.13 e Å^−3^
Δρ_min_ = −0.12 e Å^−3^

### 

Data collection: *SMART* (Bruker, 2008 ▶); cell refinement: *SAINT* (Bruker, 2008 ▶); data reduction: *SAINT*; program(s) used to solve structure: *SHELXTL* (Sheldrick, 2008 ▶); program(s) used to refine structure: *SHELXL97* (Sheldrick, 2008 ▶); molecular graphics: *PLATON* (Spek, 2009 ▶); software used to prepare material for publication: *PLATON*.

## Supplementary Material

Crystal structure: contains datablock(s) global, I. DOI: 10.1107/S1600536811025219/bv2185sup1.cif

Structure factors: contains datablock(s) I. DOI: 10.1107/S1600536811025219/bv2185Isup2.hkl

Supplementary material file. DOI: 10.1107/S1600536811025219/bv2185Isup3.cml

Additional supplementary materials:  crystallographic information; 3D view; checkCIF report

**Fig. 3.**
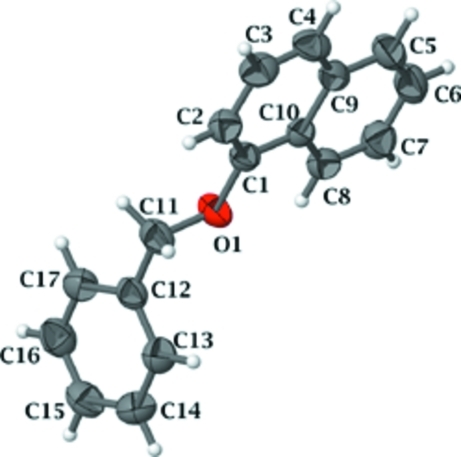
Enhanced Jmol view of the title compound showing displacement ellipsoids at the 50% probability level.

Enhanced figure: interactive version of Fig. 3

## Figures and Tables

**Table 1:** Hydrogen-bond geometry (Å, °) *Cg*1 is the centroid of the C1–C4/C9/C10 ring.

*D*—H⋯*A*	*D*—H	H⋯*A*	*D*⋯*A*	*D*—H⋯*A*
C5—H5⋯*Cg*1^i^	0.93	2.72	3.6168 (18)	161
C16—H16⋯*Cg*1^ii^	0.93	2.85	3.6847 (18)	149

Symmetry codes: (i) 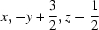; (ii) 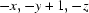.

**Fig. 1.**
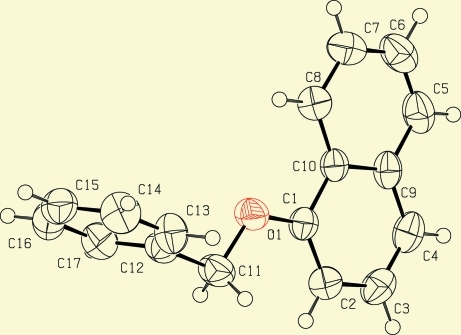
An ORTEP view of title compound showing 50% probability displacement ellipsoids.

**Fig. 2.**
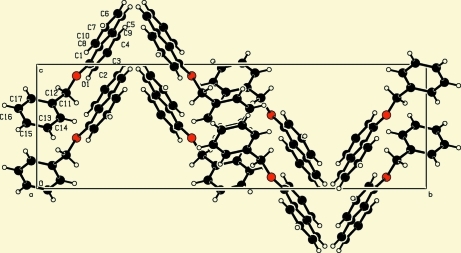
A view of packing arrangement of the titled compound along a axis.

## supplementary crystallographic information

### Comment

Benzyl groups are commonly used in organic synthesis as protecting group for alcohol, phenol, naphthol and carboxylic acids, because they are stable to acid, alkali and number of other usual reagents (Rao & Senthilkumar, 2001, He *et al.*, 2008). Benzyl ether intermediates can play an important role in sigmatropic rearrangement reactions such as Claisen and Cope rearrangements (Salunkhe *et al.*, 1994). Similarly, benzyl ethers have been used in the chemical engineering of macromolecules (Scanu *et al.*, 2007). The aromatic ether derivatives of naphthols have been used in the field of pharmaceuticals, agrochemicals and fungicides (Fernandes *et al.*, 2011). In the view of the importance of these compounds, we recently reported a simple method for the preparation of 1- naphthyl ethers (Mohamed & Arunadevi, 2010). In this connection, the crystal structure of the title compound is reported here.

In the crystal structure of the title compound (Fig. 1), both the naphthyl ring and phenyl ring are planar with a mean deviation from the least- squares plane defined by naphthyl ring carbon atoms (C1—C10) and O1 of 0.0176(0.001) Å. Similarly the mean deviation for the phenyl ring carbon atoms (C11—C16) and C17 of the benzyl group is 0.024(0.0013) Å. The dihedral angle between both planes is 83.22(0.04) °.

The angle between three atoms C1—O1—C11 is 117.72 (11) ° is in agreement with the corresponding value other structurally characterized benzyl-1-naphthyl ethers (Hassan *et al.*, 2008*a*, 2008*b*, 2008*c*, 2008d, 2009*a*, 2009*b*). However, the C1—O1 bond length is 1.3678 (17) Å, shorter compared to the same. The naphthyl ring and phenyl ring are mutually perpendicular with each other, the torsion angle between C1—O1—C11—C12 is 177.98 (12) °. The crystal structure is stabilized by weak C—H···π interactions, between the C—H(5) and centroid of the ring 1 (C1—C4/C9/C10) with the distance 2.72 Å [symmetry code: *x*, 3/2 - *y*, -1/2 + *z*] and another weak C—H···π interaction could be seen between the C—H(16) and centroid of the ring 1 (C1—C4/C9/C10) with the distance 2.85 Å [symmetry code: 1 - *x*,-*y*, 2 - *z*]. The packing diagram of the titled compound are shown in the Fig.2. The centroid to centroid π–π interactions could be observed between the phenyl ring (C12—C17) with a distance of 3.7817 (10) Å [symmetry code: -*x*, 1-*y*, -*z*].

### Experimental

A mixture of 1-naphthol (1.44 g, 10 mmol), triethylamine (1.01 g, 10 mmol) and benzyl bromide (1.71 g, 10 m.mol) in a micellar medium (10 ml) were stirred for 30 minutes at 30 °C and kept overnight at room temperature. The solid product obtained was filtered, washed with water and recrystallized from ethanol to get benzyl-1- naphthyl ether (70%, mp 354 K). The product was characterized by spectral data and compared with pervious report (Mohamed & Arunadevi, 2010).

### Refinement

All H atoms were positioned geometrically and refined using a riding model, with C—H=0.93Å for naphthyl and phenyl H atom, 0.97Å for methylene H atoms, respectively, *U*_iso_(H)= 1.2*U*_eq_(C).

### Crystal data

**Table:**

C_17_H_14_O	*F*(000) = 992
*M**_r_* = 234.28	*D*_x_ = 1.209 Mg m^−^^3^
Orthorhombic, *P**c**c**n*	Mo *K*α radiation, λ = 0.71073 Å
Hall symbol: -P 2ab 2ac	Cell parameters from 2417 reflections
*a* = 13.0210 (6) Å	θ = 3.1–21.2°
*b* = 24.8832 (10) Å	µ = 0.07 mm^−^^1^
*c* = 7.9478 (3) Å	*T* = 296 K
*V* = 2575.12 (19) Å^3^	Prism, colourless
*Z* = 8	0.07 × 0.06 × 0.06 mm

### Data collection

**Table:**

Bruker SMART CCD area-detector diffractometer	1609 reflections with *I* > 2σ(*I*)
Radiation source: sealed tube	*R*_int_ = 0.059
graphite	θ_max_ = 25.6°, θ_min_ = 1.6°
φ and ω scans	*h* = −15→14
22084 measured reflections	*k* = −30→30
2418 independent reflections	*l* = −9→9

### Refinement

**Table:**

Refinement on *F*^2^	H-atom parameters constrained
Least-squares matrix: full	*w* = 1/[σ^2^(*F*_o_^2^) + (0.0378*P*)^2^ + 0.3925*P*] where *P* = (*F*_o_^2^ + 2*F*_c_^2^)/3
*R*[*F*^2^ > 2σ(*F*^2^)] = 0.039	(Δ/σ)_max_ = 0.001
*wR*(*F*^2^) = 0.099	Δρ_max_ = 0.13 e Å^−^^3^
*S* = 1.02	Δρ_min_ = −0.12 e Å^−^^3^
2418 reflections	Extinction correction: *SHELXL97* (Sheldrick, 2008), Fc^*^=kFc[1+0.001xFc^2^λ^3^/sin(2θ)]^-1/4^
164 parameters	Extinction coefficient: 0.0079 (9)
0 restraints	

### Special details

**Table:**

Geometry. All e.s.d.'s (except the e.s.d. in the dihedral angle between two l.s. planes) are estimated using the full covariance matrix. The cell e.s.d.'s are taken into account individually in the estimation of e.s.d.'s in distances, angles and torsion angles; correlations between e.s.d.'s in cell parameters are only used when they are defined by crystal symmetry. An approximate (isotropic) treatment of cell e.s.d.'s is used for estimating e.s.d.'s involving l.s. planes.

### Fractional atomic coordinates and isotropic or equivalent isotropic displacement parameters (Å^2^)

**Table:**

	*x*	*y*	*z*	*U*_iso_*/*U*_eq_	
O1	0.03144 (8)	0.60213 (4)	0.09247 (13)	0.0529 (3)	
C1	−0.02611 (11)	0.63577 (5)	−0.00569 (18)	0.0420 (4)	
C10	0.02903 (11)	0.65924 (5)	−0.14237 (18)	0.0399 (4)	
C9	−0.02477 (12)	0.69375 (5)	−0.25359 (19)	0.0464 (4)	
C12	0.05778 (12)	0.53982 (6)	0.31348 (18)	0.0473 (4)	
C8	0.13464 (12)	0.65015 (6)	−0.16880 (19)	0.0490 (4)	
H8	0.1703	0.6273	−0.0972	0.059*	
C5	0.02988 (15)	0.71737 (7)	−0.3876 (2)	0.0621 (5)	
H5	−0.0045	0.7397	−0.4626	0.075*	
C2	−0.12767 (12)	0.64702 (6)	0.0187 (2)	0.0507 (4)	
H2	−0.1625	0.6321	0.1096	0.061*	
C13	0.12458 (13)	0.56037 (7)	0.4314 (2)	0.0595 (5)	
H13	0.1219	0.5967	0.4581	0.071*	
C11	−0.01908 (12)	0.57545 (7)	0.2298 (2)	0.0577 (5)	
H11A	−0.0763	0.5543	0.1884	0.069*	
H11B	−0.0452	0.6017	0.3093	0.069*	
C4	−0.13007 (13)	0.70384 (6)	−0.2253 (2)	0.0568 (5)	
H4	−0.1658	0.7264	−0.2981	0.068*	
C3	−0.17964 (13)	0.68115 (6)	−0.0938 (2)	0.0569 (5)	
H3	−0.2491	0.6882	−0.0775	0.068*	
C6	0.13163 (16)	0.70813 (7)	−0.4092 (2)	0.0667 (5)	
H6	0.1661	0.7242	−0.4984	0.08*	
C17	0.06300 (13)	0.48573 (7)	0.2763 (2)	0.0556 (4)	
H17	0.0183	0.4711	0.1973	0.067*	
C16	0.13394 (14)	0.45329 (7)	0.3553 (2)	0.0618 (5)	
H16	0.137	0.4169	0.329	0.074*	
C14	0.19522 (13)	0.52779 (7)	0.5101 (2)	0.0660 (5)	
H14	0.2399	0.5422	0.5894	0.079*	
C7	0.18510 (13)	0.67464 (7)	−0.2986 (2)	0.0600 (5)	
H7	0.2551	0.669	−0.3134	0.072*	
C15	0.19991 (13)	0.47420 (7)	0.4721 (2)	0.0608 (5)	
H15	0.2476	0.4522	0.5252	0.073*	

### Atomic displacement parameters (Å^2^)

**Table:**

	*U*^11^	*U*^22^	*U*^33^	*U*^12^	*U*^13^	*U*^23^
O1	0.0472 (6)	0.0611 (7)	0.0503 (7)	0.0055 (5)	0.0024 (5)	0.0189 (5)
C1	0.0438 (9)	0.0394 (8)	0.0427 (8)	0.0022 (7)	−0.0049 (7)	0.0000 (7)
C10	0.0463 (9)	0.0340 (7)	0.0394 (8)	−0.0015 (7)	−0.0037 (7)	−0.0035 (6)
C9	0.0608 (11)	0.0349 (8)	0.0434 (9)	0.0011 (7)	−0.0083 (8)	−0.0024 (7)
C12	0.0473 (9)	0.0524 (9)	0.0424 (9)	−0.0019 (8)	0.0040 (7)	0.0094 (8)
C8	0.0501 (10)	0.0525 (9)	0.0445 (9)	−0.0001 (8)	−0.0032 (8)	−0.0007 (7)
C5	0.0854 (14)	0.0488 (10)	0.0521 (10)	0.0012 (9)	−0.0036 (10)	0.0101 (8)
C2	0.0482 (10)	0.0507 (9)	0.0532 (10)	0.0005 (8)	0.0013 (8)	0.0004 (8)
C13	0.0669 (12)	0.0488 (9)	0.0627 (11)	−0.0032 (9)	−0.0085 (10)	0.0010 (9)
C11	0.0535 (11)	0.0673 (11)	0.0523 (10)	0.0006 (8)	0.0067 (8)	0.0184 (9)
C4	0.0632 (12)	0.0502 (9)	0.0570 (11)	0.0143 (8)	−0.0140 (9)	0.0010 (8)
C3	0.0462 (10)	0.0580 (10)	0.0666 (11)	0.0103 (8)	−0.0060 (9)	−0.0043 (9)
C6	0.0868 (15)	0.0621 (11)	0.0513 (11)	−0.0133 (10)	0.0093 (10)	0.0073 (9)
C17	0.0624 (11)	0.0582 (11)	0.0461 (9)	−0.0054 (9)	−0.0026 (8)	0.0010 (8)
C16	0.0750 (12)	0.0508 (10)	0.0597 (11)	0.0070 (9)	0.0035 (10)	0.0013 (9)
C14	0.0633 (12)	0.0729 (12)	0.0619 (12)	−0.0047 (10)	−0.0169 (10)	0.0001 (10)
C7	0.0559 (10)	0.0680 (11)	0.0560 (11)	−0.0078 (9)	0.0076 (9)	−0.0022 (9)
C15	0.0571 (11)	0.0673 (12)	0.0578 (11)	0.0126 (9)	0.0010 (9)	0.0132 (9)

### Geometric parameters (Å, °)

**Table:**

O1—C1	1.3677 (16)	C13—C14	1.377 (2)
O1—C11	1.4372 (17)	C13—H13	0.93
C1—C2	1.366 (2)	C11—H11A	0.97
C1—C10	1.4272 (19)	C11—H11B	0.97
C10—C8	1.409 (2)	C4—C3	1.352 (2)
C10—C9	1.4176 (19)	C4—H4	0.93
C9—C5	1.409 (2)	C3—H3	0.93
C9—C4	1.412 (2)	C6—C7	1.397 (2)
C12—C13	1.377 (2)	C6—H6	0.93
C12—C17	1.380 (2)	C17—C16	1.378 (2)
C12—C11	1.493 (2)	C17—H17	0.93
C8—C7	1.367 (2)	C16—C15	1.367 (2)
C8—H8	0.93	C16—H16	0.93
C5—C6	1.356 (2)	C14—C15	1.369 (2)
C5—H5	0.93	C14—H14	0.93
C2—C3	1.407 (2)	C7—H7	0.93
C2—H2	0.93	C15—H15	0.93
			
C1—O1—C11	117.72 (12)	O1—C11—H11B	110.1
C2—C1—O1	125.11 (14)	C12—C11—H11B	110.1
C2—C1—C10	120.74 (13)	H11A—C11—H11B	108.5
O1—C1—C10	114.15 (12)	C3—C4—C9	120.82 (15)
C8—C10—C9	119.11 (14)	C3—C4—H4	119.6
C8—C10—C1	122.59 (13)	C9—C4—H4	119.6
C9—C10—C1	118.29 (13)	C4—C3—C2	120.93 (16)
C5—C9—C4	122.44 (15)	C4—C3—H3	119.5
C5—C9—C10	118.31 (15)	C2—C3—H3	119.5
C4—C9—C10	119.24 (14)	C5—C6—C7	120.58 (16)
C13—C12—C17	118.49 (15)	C5—C6—H6	119.7
C13—C12—C11	120.38 (15)	C7—C6—H6	119.7
C17—C12—C11	121.12 (15)	C16—C17—C12	120.45 (16)
C7—C8—C10	120.67 (15)	C16—C17—H17	119.8
C7—C8—H8	119.7	C12—C17—H17	119.8
C10—C8—H8	119.7	C15—C16—C17	120.49 (16)
C6—C5—C9	121.22 (16)	C15—C16—H16	119.8
C6—C5—H5	119.4	C17—C16—H16	119.8
C9—C5—H5	119.4	C15—C14—C13	120.21 (16)
C1—C2—C3	119.97 (15)	C15—C14—H14	119.9
C1—C2—H2	120	C13—C14—H14	119.9
C3—C2—H2	120	C8—C7—C6	120.08 (17)
C14—C13—C12	120.84 (15)	C8—C7—H7	120
C14—C13—H13	119.6	C6—C7—H7	120
C12—C13—H13	119.6	C16—C15—C14	119.51 (16)
O1—C11—C12	107.80 (12)	C16—C15—H15	120.2
O1—C11—H11A	110.1	C14—C15—H15	120.2
C12—C11—H11A	110.1		
			
C11—O1—C1—C2	1.5 (2)	C11—C12—C13—C14	179.53 (15)
C11—O1—C1—C10	−178.07 (12)	C1—O1—C11—C12	177.97 (12)
C2—C1—C10—C8	177.82 (14)	C13—C12—C11—O1	83.11 (18)
O1—C1—C10—C8	−2.56 (19)	C17—C12—C11—O1	−97.56 (17)
C2—C1—C10—C9	−1.1 (2)	C5—C9—C4—C3	−178.92 (15)
O1—C1—C10—C9	178.56 (12)	C10—C9—C4—C3	0.2 (2)
C8—C10—C9—C5	0.5 (2)	C9—C4—C3—C2	0.2 (2)
C1—C10—C9—C5	179.38 (13)	C1—C2—C3—C4	−1.0 (2)
C8—C10—C9—C4	−178.67 (13)	C9—C5—C6—C7	0.2 (3)
C1—C10—C9—C4	0.3 (2)	C13—C12—C17—C16	−0.3 (2)
C9—C10—C8—C7	0.7 (2)	C11—C12—C17—C16	−179.60 (15)
C1—C10—C8—C7	−178.16 (14)	C12—C17—C16—C15	0.2 (3)
C4—C9—C5—C6	178.20 (16)	C12—C13—C14—C15	−0.1 (3)
C10—C9—C5—C6	−0.9 (2)	C10—C8—C7—C6	−1.5 (2)
O1—C1—C2—C3	−178.15 (14)	C5—C6—C7—C8	1.0 (3)
C10—C1—C2—C3	1.4 (2)	C17—C16—C15—C14	−0.1 (3)
C17—C12—C13—C14	0.2 (2)	C13—C14—C15—C16	0.0 (3)

### Hydrogen-bond geometry (Å, °)

**Table:**

Cg1 is the centroid of the C1–C4/C9/C10 ring.

**Table:**

*D*—H···*A*	*D*—H	H···*A*	*D*···*A*	*D*—H···*A*
C5—H5···Cg1^i^	0.93	2.72	3.6168 (18)	161
C16—H16···Cg1^ii^	0.93	2.85	3.6847 (18)	149

Symmetry codes: (i) *x*, −*y*+3/2, *z*−1/2; (ii) −*x*, −*y*+1, −*z*.
